# Safety of vaccines administration in hereditary fructose intolerance

**DOI:** 10.1186/s13023-020-01552-z

**Published:** 2020-10-01

**Authors:** Arianna Maiorana, Antonella Sabia, Tiziana Corsetti, Carlo Dionisi-Vici

**Affiliations:** 1 Division of Metabolism, Ospedale Pediatrico Bambino Gesù, IRCSS, piazza S. Onofrio 4, 00165 Rome, Italy; 2 Hospital Pharmacy, Ospedale Pediatrico Bambino Gesù, IRCSS, Rome, Italy

**Keywords:** Hereditary fructose intolerance, Fructose, Sorbitol, Sucrose, Vaccines

## Abstract

Patients with hereditary fructose intolerance need to follow a life-long fructose dietary and drug restriction to prevent symptoms of intoxication. Concerns about vaccines administration have been manifested overtime, for the risk of a life-threatening acute intoxication. For this reason, at Ospedale Pediatrico Bambino Gesù we performed a deepen research from open sources, datasheets and Pharmaceutical Companies informations from the most common Italian and European vaccines, which are carried out in infancy and childhood. As a safe threshold of 2.4 mg/kg/dose was recently established for oral and parenteral (other than i.v.) route, the manuscript clarifies the safe administration of majority of vaccines in patients with hereditary fructose intolerance.

Hereditary fructose intolerance (HFI, OMIM 229,600) is an autosomal-recessive disorder with a prevalence of 1:23,000, caused by deficiency of aldolase B, the main enzyme responsible for hepatic metabolism of fructose. The signs and symptoms upon introduction of fructose-containing foods include abdominal pain, nausea, recurrent vomiting, hypoglycemia and failure to thrive. Besides hypoglycemia, metabolic disturbances such as lactic acidemia, hypophosphatemia, hyperuricemia and hypermagnesemia are observed in case of acute fructose intoxication. Parenteral intravenously administration of fructose, sorbitol, or sucrose may cause death for severe hypoglycemia, acute hepato-renal failure associated with bleeding and jaundice and must be rigorously avoided [1]. For this reason, the diagnostic challenge with i.v. fructose 0.25 g/kg was abandoned and substituted by the molecular analysis. Also the diagnostic oral load of fructose 1 g/kg is no longer recommended as deemed life threatening as well.

Many individuals with HFI exhibit a self-imposed aversion to sweet foods, sufficiently to prevent an acute intoxication. However, prolonged fructose intake leads to poor feeding, vomiting, failure to thrive, hepatomegaly, liver and renal tubular dysfunction that might lead to irreversible liver and kidney damage [1, 2]. Upon dietary restriction of fructose, symptoms resolve and normal growth and development are achieved. Therefore, individuals with HFI need to be treated life-long with a fructose-restricted diet. They should be aware of the presence of fructose in certain medicinal formulations and such medications should be avoided. Particularly, concerns about oral or parenteral vaccines administration have been manifested overtime, because they contain amounts of sorbitol or sucrose or other analogous sugars.

However, there is no scientifically established and generally accepted safe dose for patients with HFI despite the fact that, at least in childhood, the intake of fructose should not be determined by subjective tolerance. As a matter of fact, the spectrum of individual fructose tolerance in HFI appears to depend on age (infants being more sensitive than adults), beyond the respective gene mutation [3].

Scientific evidences are very poor. One paper advises that short-term (2 days) oral administration of 4.7 mg/kg/day of fructose was safe and well tolerated among 5 individuals (aged 14–52 years) with diagnosed HFI [4], with normal blood chemistry and only a slight elevation of uric acid in two patients. An internationally accepted safety recommendation restricts the oral fructose intake below 40 mg/kg/day, whereas the more stringent fructose restriction reported is 10 mg/kg/day [1, 5]. This level of 10 mg/kg/day was chosen from the European Medicine Agency (EMA) [5] for the calculation of a threshold to generate informations for a package leaflet regarding fructose and sorbitol used as excipients in medicinal products for human oral use. This conservative approach was chosen as the intake from a medicinal product will always be additive to the dietary intake. Although at this level the intestinal absorption capacity (0.5 g/kg) is probably not reached, there are no data on PK from subcutaneous or intramuscular absorption of fructose and sorbitol. However, it can be assumed that parenteral administration might be faster and consequently plasma concentration might be higher. Therefore, the orally tolerable dose of 10 mg/kg/day was divided by a factor of 2 to be safely applicable also for s.c/i.m route, expecting for a parenteral (other than i.v.) dose of 5 mg/kg/day a similar systemic exposure level (Cmax and AUC) than an oral fructose intake of 10 mg/kg/day [5].

Thus, for all oral and parenteral (other than i.v., for which the threshold is zero) products below the threshold of 5 mg/kg/day it has been proposed to declare the sorbitol content but not to include a warning [5]. This threshold is not regarded as a safe daily dose but as a limit above which a detailed warning in the leaflet is deemed necessary and useful. Indeed, above this threshold the warning remained for all administration routes. Nonetheless, in some vaccines a warning within the datasheet states “Do not administer in subjects with HFI”, even when the content of sorbitol or sucrose does not exceed this limit.

The need to avoid any hazardous exposure to fructose contained in vaccines collides with the need of immunization for the most vulnerable age group from severe infections. Furthermore, some vaccines have been administered for a long time without any incidence of severe events due to HFI, for instance in patients with a delayed diagnosis. For these reasons, at Ospedale Pediatrico Bambino Gesù of Rome, we performed a deepen research from open sources (Pubmed, Cochrane) and datasheets available from the most common vaccines which are carried out in infancy (0–1 years), early (2–5 years) and mid childhood (6–11 years) in Italy and in the other European Countries where vaccines have the same international authorization commerce (Table 1). We collaborated with our hospital Pharmacy and the Pharmaceutical Companies to outline the exact amount of fructose, sucrose, sorbitol and other fructose analogues when not reported in the datasheets. We performed a deep research through the open sources in Italian databases Codifa (https://www.codifa.it) and AIFA (https://www.agenziafarmaco.gov.it/content/vaccini), and in the European Medicines Agency (https://www.ema.europa.eu/en/medicines).

**Table 1:** Types of vaccines and their European distribution

Vaccine name	Vaccine components	Fructose analogues dose	Marketing authorization holder	Member State where product is authorised
Antihaemophilus B vaccine				
ACTHIB-intramuscular or subcutaneous use	ANTIHAEMOPHILUS B	Sucrose	Sanofi Pasteur	IT
HIBERIX-intramuscular use	ANTIHAEMOPHILUS B	None	GlaxoSmithKline	IT
The antihaemophilus B component is also present in the following vaccines				
*HEXYON *INFANRIX HEXA				
Antihepatitis vaccine B				
ENGERIX B-intramuscular use	ANTIHEPATITIS B	None	GlaxoSmithKline	BG, DE, DK, EL, ES, FR, IT, NL, PT, SE
HBVAXPRO-intramuscular use	ANTIHEPATITIS B	None	Sanofi Pasteur	BG, CZ, DE, DK, EE, EL, ES, FI, FR, HU, IT, LT, LV, MT, NL, PT, RO, SE, SI, SK
The hepatitis B component is also present in the following vaccines				
TWINRIX adults intramuscular use	ANTIHEPATITIS B—ANTIHEPATITIS A	None	GlaxoSmithKline	BG, CZ, DE, DK, EE, EL, ES, FI, FR, HU, IT, LV, LT, MT, NL, PL, PT, RO, SE, SI, SK
TWINRIX pediatric)-intramuscular use	ANTIHEPATITIS B—ANTIHEPATITIS A	None	GlaxoSmithKline	BG, CZ, DE, DK, EE, EL, ES, FI, FR, HU, IT, LT, LV, MT, NL, PL, PT, RO, SE, SI, SK
The hepatitis B component is also present in the following vaccines				
*HEXYON *INFANRIX HEXA				
Antimeasles vaccine				
PRIORIX-subcutaneous use	ANTIMEASLES—ANTIRUBELLA—ANTIMUMPS	Sorbitol 9 mg Mannitol	GlaxoSmithKline	BG, CZ, DE, DK, EE, EL, ES, FI, FR, HU, IT, LT, LV, MT, NL, PL, PT, RO, SE, SI, SK
M-M-RVAXPRO-intramuscular or subcutaneous use	ANTIMEASLES—ANTIRUBELLA—ANTIMUMPS	Sorbitol 14.5 mg Polysorbate 80 contained in the medium excipient 199^§^	MSD Vaccines	AT, BE, BG, CY, CZ, DE, DK, EE, EL, ES, FI, FR, IE, IS, IT, HR, HU, LT, LU, LV, MT, NL, NO, PL, PT, RO, SE, SI, SK, UK
The anti-measles component is also present in the following vaccines				
*HEXYON *INFANRIX HEXA				
Antimeningococcal B vaccine				
BEXSERO-intramuscular use	ANTI-MENINGOCOCCAL B	Sucrose	GlaxoSmithKline	BG, CZ, DE, DK, EE, EL, ES, FI, FR, HR, HU, IT, LT, LV, MT, NL, PL, PT, RO, SE, SK
TRUMENBA-intramuscular use	ANTI-MENINGOCOCCAL B	Polysorbate 80	Pfizer Europe	BG, CZ, DE, DK, EE, EL, ES, FI, FR, HR, HU, IT, LT, LV, MT, PL, PT, RO, SE, SK
Antimeningococcal C vaccine				
MENJUGATE-intramuscular use	ANTI-MENINGOCOCCAL C	None	GlaxoSmithKline	IT
NEISVAC-C-intramuscular use	ANTI-MENINGOCOCCAL C	Sucrose	Pfizer S.r.l	IT
The anti-meningococcal component C is also present in the following vaccines				
MENVEO-intramuscular use	CONJUGATED ANTI-MENINGOCOCCUS A, C, W135, Y	Sucrose	GlaxoSmithKline	BG, CZ, DE, DK, EE, EL, ES, FI, FR, HR, HU, IT, LT, LV, MT, NL, PL, PT, RO, SE, SK
NIMENRIX-intramuscular use	CONJUGATED ANTI-MENINGOCOCCUS A, C, W135, Y	Sucrose	Pfizer S.r.l	BG, CZ, DE, DK, EE, EL, ES, FI, FR, HR, HU, IT, LT, LV, MT, NL, PL, PT, RO, SE, SI, SK,
Antipapilloma virus				
GARDASIL 9-intramuscular use	HUMAN ANTIPAPILLOMAVIRUS (human types 6, 11, 16, 18, 31, 33, 45, 52, 58)	Polysorbate 80	MSD Vaccines	AT, BE, BG, CZ, CY, DE, DK, EE, EL, ES, FI, FR, HU, IE, IS, IT, LT, LU, LV, MT, NL, NO, PL, PT, RO, SE, SI, SK, UK
Antipneumococcal vaccine				
PNEUMOVAX-intramuscular or subcutaneous use	ANTI-PNEUMOCOCCICAL	none	Sanofi Pasteur	IT
PREVENAR 13-intramuscular use	ANTI-PNEUMOCOCCICAL	Polysorbate 80	Pfizer S.r.l	BG, CZ, DE, DK, EE, EL, ES, FI, FR, HR, HU, IT, LT, LV, MT, NL, PL, PT, RO, SE, SK
SYNFLORIX-intramuscular use	ANTI-PNEUMOCOCCICAL	none	GlaxoSmithKline	BG, CZ, DE, DK, EE, EL, ES, FI, FR, HR, HU, IT, LT, LV, MT, NL, PL, PT, RO, SE, SK
Antipolio vaccine				
IMOVAX POLIO-intramuscular or subcutaneous use	ANTIPOLIO	None	Sanofi Pasteur	IT
The polio component is also present in the following vaccines				
HEXYON-intramuscular use	ANTIPOLIO—ANTIDIPHTERIA—ANTITETANUS—ANTIPERTUSSIS—ANTIHAEMOPHILUS B—ANTIHEPATITIS B	Saccarose 10.6 mg	Sanofi Pasteur	AT, BE, BG, CY, CZ, DE, DK, EE, EL, ES, FI, FR, HR, HU, IE, IS, IT, LT, LU, LV, MT, NL, NO, PL, PT, RO, SI, SK, SE, UK
INFANRIX HEXA-intramuscular use	ANTIPOLIO—ANTIDIPHTERIA—ANTITETANUS—ANTIPERTUSSIS—ANTIHAEMOPHILUS B—ANTIHEPATITIS B	Polysorbate 80 contained in the medium excipient 199	GlaxoSmithKline	BG, CZ, DE, DK, EE, EL, ES, FI, FR, HR, HU, IS, IT, LT, LV, MT, NL, NO, RO, SE, SI, SK
POLIOBOOSTRIX intramuscular use	ANTIPOLIO—ANTIDIPHTERIA—ANTITETANUS—ANTIPERTUSSIS	Polysorbate 80 contained in the medium excipient 199	GlaxoSmithKline	AT, BG, DE, EL, ES, FI, FR, HR, HU, IE, IS, IT, LT, LU, LV, MT, NL, NO, PL, PT, RO, SE, SI, SK, UK
POLIOINFANRIX intramuscular use	ANTIPOLIO—ANTIDIPHTERIA—ANTITETANUS—ANTIPERTUSSIS	Polysorbate 80 contained in the medium excipient 199	GlaxoSmithKline	BE, DE, EE, EL, FI, FR, HR, HU, IE, IT, LT, LU, LV, MT, NL, NO, PL, PT, SE, SK, UK
REVAXIS-intramuscular or subcutaneous use	ANTIPOLIO—ANTIDIPHTERIA—ANTITETANUS	Polysorbate 80 contained in the medium excipient 199	Sanofi Pasteur	AT, BE, DE, ES, FR, IE, IT, LU, NL, PT, UK
TRIAXIS POLIO—intramuscular use	ANTIPOLIO—ANTIDIPHTERIA—ANTITETANUS—ANTIPERTUSSIS	Polysorbate 80 contained in the medium excipient 199	Sanofi Pasteur	IT
TETRAVAC-intramuscular use	ANTIPOLIO—ANTIDIPHTERIA—ANTITETANUS—ANTIPERTUSSIS	Polysorbate 80 contained in the medium excipient 199	Sanofi Pasteur	AT, BE, DE, DK, EE, EL, FI, FR, IE, IS, IT, LU, LV, NO, PT, SE, UK
VAXELIS-intramuscular use	ANTIPOLIO—ANTIDIPHTERIA—ANTITETANUS—ANTIPERTUSSIS—ANTIHAEMOPHILUS B—ANTIHEPATITIS B	None	MCM Vaccine	BG, CZ, DE, DK, EE, EL, ES, FI, FR, HR, HU, IT, LT, LV, MT, PL, PT, RO, SE, SI, SK
Antirotavirus vaccine				
ROTATEQ-oral use	ANTIROTAVIRUS pentavalent, live, reassorting	Sucrose 1,080 mg Polysorbate 80	Sanofi Pasteur	BG, CZ, DE, DK, EE, EL, ES, FI, FR, HR, HU, IT, LT, LV, MT, NL, PL, PT, RO, SE, SI, SK
ROTARIX-oral suspension	ANTIROTAVIRUS live, attenuated	Sucrose 1.073 mg	GlaxoSmithKline	BG, CZ, DE, DK, EE, EL, ES, FI, FR, HR, HU, IT, LT, LV, MT, NL, PL, PT, RO, SE, SI, SK
ROTARIX-powder and solvent for oral suspension	ANTIROTAVIRUS live, attenuated	Sucrose 9 mg Sorbitol 13.5 mg	GlaxoSmithKline	BG, CZ, DE, DK, EE, EL, ES, FI, FR, HR, HU, LT, LV, MT, NL, PL, PT, RO, SE, SI, SK
Antitetan vaccine				
ANATETALL-intramuscular use	ANTITETANUS adsorbed	None	GlaxoSmithKline	DE, HU, IT, SI
IMOVAX TETANO-intramuscular or subcutaneous use	ANTITETANUS	None	Sanofi Pasteur	CZ, DE, FR, HR, IT, MT, NO, RO, SK
The antitetan component is also present in the following vaccines				
BOOSTRIX-intramuscular use	ANTITETANUS—ANTIDIPHTERIA—ANTIPERTUSSIS	None	GlaxoSmithKline	AT, BE, BG, CY, DE, DK, EL, ES, FI, HR, HU, IE, IS, IT, LT, LU, LV, MT, NL, NO, PL, PT, SE, SI, SK, UK
DIFTETALL-intramuscular use	ANTITETANUS—ANTIDIPHTERIA	None	Astro-Pharma	IT
INFANRIX DTPA-intramuscular use	ANTITETANUS—ANTIDIPHTERIA—ANTIPERTUSSIS	Polysorbate 80	GlaxoSmithKline	IT
TRIAXIS-intramuscular use	ANTITETANUS—ANTIDIPHTERIA—ANTIPERTUSSIS	None	Sanofi Pasteur	BE, DK, EL, ES, FI, FR, IE, IS, LU,SE, NL, NO
TRIBACCINE-intramuscular use	ANTITETANUS—ANTIDIPHTERIA—ANTIPERTUSSIS	None	AJ Vaccines A/S	IT
*HEXYON *INFANRIX HEXA *POLIOBOOSTRIX *POLIOINFANRIX *REVAXIS *TETRAVAC				
Antivaricella vaccine				
VARILRIX-subcutaneous use	ANTICHICKENPOX	Sorbitol 6 mg Mannitol	GlaxoSmithKline	IT
VARIVAX-intramuscular or subcutaneous use	ANTICHICKENPOX	Sucrose	MSD Italia S.r.l	IT
The antivaricella component is also present in the following vaccines				
PRIORIX TETRA subcutaneous use	ANTICHICKENPOX—ANTIMEASLES—ANTIRUBELLA—ANTIMUMPS	Sorbitol 14 mg Mannitol Polysorbate 80 contained in the medium excipient 199	GlaxoSmithKline	BG, CZ, DE, DK, EE, EL, ES, FI, FR, HU, IT, LT, LV, MT, NL, PL, PT, RO, SE, SI, SK
PROQUAD-intramuscular or subcutaneous use	ANTICHICKENPOX—ANTIMEASLES—ANTIRUBELLA—ANTIMUMPS	Sorbitol 16 mg Sucrose Polysorbate 80 contained in the medium excipient 199	MSD Italia S.r.l	BG, CZ, DE, DK, EE, EL, ES, FI, FR, HU, IT, LT, LV, MT, NL, PL, PT, RO, SE, SI, SK

The table displays the different types of vaccines which are commercialized in Europe and their amount of fructose analogues content

*AT* Austria, *BE* Belgium BE, *BG* Bulgary, *CY* Cyprus, *CZ* Czech Republic, *DE* Germany, *DK* Denmark, *EE* Estonia, *EL* Greece, *ES* Spain, *FI* Finland, *FR* France, *HR* Croatia, *IE* Ireland, *IS* Iceland, *LT* Lithuania, *LU* Luxembourg, *HU* Hungary, *MT* Malta, *NL* Netherland, *NO* Norway, *PL* Poland, *PT* Portugal, *RO* Romania, *SI* Slovenia, *SK* Slovakia, *IT* Italy, *LV* Latvia, *SE* Sweden, *UK* United Kingdom

^§^Medium excipient 199, a complex of amino acids, mineral salts, vitamins, polysorbate 80 and other substances diluted in water for injections

We obtained the following informations:

- Rotarix pre-established oral suspension (clear and colourless liquid, the only formulation commercially available in Italy) and Rotateq contain sucrose above 1000 mg/dose, therefore they are contraindicated in subjects with HFI
- Rotarix white powder and solvent for oral suspension, commercially available in other European Countries, containing sucrose 9 mg and sorbitol 13.5 mg, can be administered in HFI children with a weight > 9.3 kg (2.4 mg/kg/dose)
- Imovax polio, Vaxelis, Engerix B, HBVAXPRO, Twinrix, Hiberix, Menjugate, Pneumovax, Synflorix, Anatetall, Imovax tetano, Boostrix, Diftetall, Triaxis, Tribaccine do not contain fructose nor analogues sugars, therefore they can be safely administrated in subjects with HFI
- M-M-RVAXPRO, Proquad, Varilrix, Hexyon, Priorix and Priorix tetra contain sorbitol up to 16 mg + sucrose or mannitol traces, therefore, according to a recent document of Istituto Superiore di Sanità of Italy [6], they can be administered with reasonable safety, whenever a threshold of 2.4 mg/kg/dose will not be exceeded (assumption: lower than 3rd percentile of weight-for-age in females at 9 months: 6.6 kg according to WHO Multicentre Study Growth Reference Study Group, 2006). Thus, in accordance with the Italian vaccinal calendar, the administration of M-M-RVAXPRO, Proquad and Varilrix can be done despite the warning in the datasheet stating “Do not administer in subjects with HFI”
- All other vaccines contain fructose and analogues in traces up to 10 mg. Therefore, according to the above recommendations [6] they can be administered with reasonable safety from infancy. Furthermore, the monoclonal antibody Palivizumab (Synagis, AbbVie), used for Respiratory Syncytial Virus’ immunization, contains mannitol traces and can be safely administered in preterm newborns.

In summary, according to the recommendation of Istituto Superiore di Sanità of Italy [6], considering a limit of 2.4 mg/kg/dose as a safe threshold for oral and parenteral (s.c./i.m.) route, majority of vaccines can be safely administered in infants and children with HFI. The vaccines M-M-RVAXPRO and Proquad can be administered after 9 months at a weight > 6.5 kg. Rotarix white powder and solvent for oral suspension can be administered at a weight > 9.3 kg. The only contraindicated vaccines in HFI are Rotarix pre-established oral suspension and Rotateq.

## Data Availability

All data generated or analysed during this study are included in this published article.

## Abbreviations

AIFA: Italian Agency of Drug
AUC: Area under the curve
Cmax: Maximum concentration
EMA: European Medicine Agency
HFI: Hereditary fructose intolerance
i.m.: Intramuscular
i.v.: Intravenous
PK: Pharmacokinetics
s.c.: Subcutaneous
WHO: World Health Organization
